# Physical activity participation and obesity-related risk behaviors among male Saudi adolescents: a social cognitive theory perspective

**DOI:** 10.3389/fpubh.2026.1885913

**Published:** 2026-07-27

**Authors:** Abdulrahman I. Alaqil

**Affiliations:** College of Education, Department of Physical Education, King Faisal University, Al-Ahsa, Saudi Arabia

**Keywords:** adolescents, obesity, physical activity, Saudi Arabia, screen time, social cognitive theory, waist-to-height ratio

## Abstract

**Background:**

Compounded by physical inactivity and an obesogenic environment, adolescent obesity represents a significant public health challenge for Saudi Arabia. Drawing on Social Cognitive Theory (SCT) as a conceptual lens, this study aimed to compare obesity-related risk behaviors among physically active and physically inactive male Saudi adolescents.

**Method:**

Using a cross-sectional design and convenience sampling, male Saudi high school students (*N* = 124) aged 16–17 years in Riyadh were classified into physically active (*n* = 70; self-reported vigorous-intensity activity or structured ≥3 days/week) and physically inactive (*n* = 54) participants. BMI was classified according to International Obesity Task Force (IOTF) cutoffs, and waist-to-height ratio (WHtR) defined abdominal obesity. Lifestyle behaviors (PA, screen time, sleep, and diet) were assessed using the Arab Teen Lifestyle Study (ATLS) questionnaire.

**Results:**

Physically inactive participants had a significantly higher BMI than physically active peers (30.28 vs. 23.64 kg/m^2^; *p* < 0.001; *d* = 1.060), waist circumference (97.35 vs. 85.46 cm; *p* < 0.001; *d* = 0.866), and WHtR (0.562 vs. 0.488; *p* < 0.001; *d* = 0.927). By IOTF criteria, 51.9% of physically inactive participants were obese versus 17.1% of physically active participants, and 72.2% exhibited abdominal obesity (WHtR ≥ 0.50) versus 31.4%. Physically active participants accumulated significantly greater METs-min/week (4,083.9 vs. 2,225.0; *p* < 0.001; *d* = 0.667) and PA minutes/week (795.6 vs. 421.9; *p* < 0.001; *d* = 0.758); 68.6% were highly active versus 24.1% of physically inactive participants, while 63.0% of physically inactive participants were sedentary. Screen time and sleep did not differ significantly between groups; insufficient sleep was prevalent in both groups (84.3% vs. 83.3%). Of ten dietary behaviors assessed, only fresh fruit consumption was significantly higher among physically active participants (5.44 vs. 4.24 days/week; *p* = 0.006; *d* = 0.520).

**Conclusion:**

Physically active was associated with lower obesity risk in male Saudi adolescents, with large between-group differences in BMI, WHtR, and abdominal obesity. Unsatisfactory screen time and sleep profiles across both groups suggest a pervasive obesogenic environment irrespective of PA status. These cross-sectional findings highlight the potential value of PA promotion, dietary reforms, and screen time and sleep initiatives for this population.

## 1 Introduction

Adolescent obesity represents a global public health emergency, with prevalence more than doubled over the past three decades and all projections indicating continued escalation (1, 2). Adolescence is a critical period during which obesogenic behaviors (e.g., physical inactivity, poor diet, excessive sedentary time, and inadequate sleep) are established and consolidated, with serious consequences that extend into adulthood (3–5). In the Gulf Cooperation Council (GCC) countries, the burden of adolescent obesity is particularly severe. In 2023, the World Health Organization (WHO) reported that among school-age children and adolescents (aged 5–19), GCC countries recorded the highest prevalence of overweight globally in 2016, with Saudi Arabia, Kuwait, Qatar, and the United Arab Emirates each reporting adult obesity rates more than double the global average (6). Driven by rapid urbanization, economic transitions, and profound shifts in lifestyle patterns, a systematic review of school-age children and adolescents in GCC countries confirmed that the region faces one of the highest pediatric obesity burdens worldwide (3). In the case of Saudi Arabia specifically, a recent multicenter population-based study reported an obesity prevalence of 49.7% among those aged 2–19 years, foregrounded by escalating trends over the past two decades (7). Sociodemographic factors, including sex and school type, significantly modulate body mass index (BMI) and PA profiles among Saudi adolescents (8), while physical inactivity in particular has been identified as a central and modifiable driver of this trajectory (9).

Multiple obesity-related risk behaviors co-occur among physically inactive adolescents. Sedentary behavior, poor dietary habits, and insufficient sleep operate independently yet synergistically to promote adiposity and cardiometabolic dysfunction (10, 11). Understanding why adolescents do or do not engage in regular PA requires the use of a robust theoretical lens. Social Cognitive Theory (SCT), proposed by Bandura (1986), offers one of the most comprehensive and empirically validated frameworks for conceptualizing health behavior (12). SCT posits that behavior arises from the reciprocal interaction among personal cognitive factors, behavioral patterns, and environmental influences, termed reciprocal determinism (12). Its core constructs, namely self-efficacy, outcome expectations, observational learning, social support, and behavioral capability, have been consistently identified as predictors of PA engagement and obesity prevention across adolescent populations (13–15). Systematic reviews confirm that SCT-based interventions targeting these constructs produce meaningful improvements in PA engagement and weight-related outcomes in adolescents (15–17). Although the current study does not directly measure SCT constructs, SCT serves as a conceptual lens for interpreting between-group behavioral differences and informing the discussion of potential mechanisms.

Despite the substantial research on adolescent obesity in Saudi Arabia and GCC countries, a critical gap persists. Prior studies have largely described lifestyle behaviors at the population level or examined general correlates of obesity without systematically comparing the full behavioral risk profile of physically active versus physically inactive adolescents using a validated instrument (3, 7). Critically, no attempts have been made to incorporate the Arab Teens Lifestyle Study (ATLS) questionnaire, a validated, culturally appropriate tool for Arab adolescents, within a theoretically informed framework to compare obesity-related risk behaviors between physically active and physically inactive adolescents in Saudi Arabia. The novelty of the current study lies in three aspects: the use of a group-comparative design targeting the full behavioral risk profile simultaneously; the application of the ATLS in this specific comparative context; and the use of SCT as conceptual framework for interpreting behavioral differences, which advances beyond descriptive epidemiological approaches by providing a theoretically grounded basis for understanding potential mechanisms and informing intervention design. This oversight limits the evidence base for targeted interventions to encourage physically inactive Saudi adolescents to engage in PA. Accordingly, the current study aimed to compare obesity-related risk behaviors among physically active and physically inactive male Saudi adolescents, drawing on SCT as a conceptual framework.

## 2 Method

### 2.1 Study design and setting

A cross-sectional study was conducted to compare obesity-related risk behaviors among physically active and physically inactive Saudi adolescents in Riyadh, Saudi Arabia. Ethical approval was obtained from the Institutional Review Board of King Saud University (Reference: KSU-HE-22-891). All research procedures were conducted in accordance with the Declaration of Helsinki. Written informed consent was obtained from all participants; given that participants were aged 16–17, written parental or guardian consent was also secured prior to their enrolment in the study.

### 2.2 Participants and recruitment

Participants were recruited from male-only public high schools in Riyadh using convenience sampling. It should be noted that this sampling approach introduces the potential for selection bias, as adolescents attending male-only public school in Riyadh may differ systematically from those in private school, rural regions, or female populations with respect to lifestyle behavior, socioeconomic background, and obesity risk; findings should therefore be interpreted with appropriate caution regarding external validity. The sample (*N* = 124) comprised male students aged 16–17. For the purposes of group allocation, participants were classified as physically active (*n* = 70), or physically inactive (*n* = 54) based on self-reported PA participation status. Physically active participants were defined as those who self-reported engaging in vigorous-intensity physical activity (VPA) or structured physical training on 3 or more days per week, consistent with established criteria in adolescent PA research (18–21). Participants who did not meet this threshold were classified as physically inactive. It should be noted that this binary grouping criterion (≥3 days/week VPA) was the sole criterion used for group allocation and was distinct from the ATLS-based METs-min/week classification used subsequently in the PA analysis section to characterize the distribution of PA levels within the full sample. To determine the required sample size, an *a priori* power analysis conducted using G*Power (version 3.1.9.7) indicated that a sample of 124 participants was sufficient to detect a medium effect size (Cohen’s d = 0.50) with 80% statistical power at a two-tailed significance level of *α* = 0.05 for independent samples t-test, thus confirming the adequacy of the sample size.

### 2.3 Anthropometric measurements

Standing height was measured to the nearest 0.1 cm using a calibrated stadiometer (Detecto Electronic, Model DHRWM, Webb City, MO, United States). Body weight was recorded to the nearest 0.1 kg using a digital scale (Seca-869, Hamburg, Germany). Participants were measured in the morning, wearing minimal clothing and no shoes. Body mass index (BMI) was calculated as body mass in kilograms divided by height in meters squared (kg/m2) and classified according to the extended International Obesity Task Force (IOTF) age and sex-specific reference standards. The IOTF cutoffs correspond to adult BMI values of 18.5, 25, and 30 kg/m^2^ at 18 years of age for underweight, overweight, and obese, respectively (22). Combined overweight and obesity prevalence was calculated based on BMI ≥ 25 kg/m^2^.

Waist circumference (WC) was measured twice at the midpoint between the lowest rib and the iliac crest using a non-stretchable tape measure (Seca 201, Hamburg, Germany); the mean of the two measurements was used in all analyses. The waist-to-height ratio (WHtR) was subsequently calculated as WC (cm) divided by height (cm), with a cutoff of ≥ 0.50 used to define abdominal obesity, as per established recommendations (23). WHtR offers the practical advantage of not requiring population-specific or age and sex-specific reference cutoffs, unlike using WC alone.

### 2.4 Study questionnaire

Lifestyle behavior data were collected using the Arab Teen Lifestyle Study (ATLS) questionnaire, a validated, self-administered instrument comprising 47 items designed to evaluate health-related behaviors among adolescents and young adults aged 14–25 (24). The ATLS has demonstrated acceptable reliability and validity for assessing physical activity, sedentary behavior, sleep duration, and dietary habits in Arab adolescent populations and has been widely applied across Saudi Arabia and the broader MENA region (10, 25). As all behavioral data were collected via self-report, the potential for recall bias and social desirability bias should be acknowledged; this limitation applies particularly to the estimation of PA, dietary intake, sleep duration, and screen time, and is discussed further in the limitation section.

### 2.5 Physical activity

PA data was collected using the ATLS questionnaire. This included the frequency, duration, and perceived intensity of physical activities performed during a typical week across multiple domains, including walking, moderate-intensity activity, vigorous-intensity activity, and household chores. PA was categorized by intensity: light, moderate, or vigorous. Total weekly PA energy expenditure was calculated in METs per minute per week (METs-min/week) by multiplying the MET intensity value of each activity by the total time spent engaged in that activity (minutes/week), summed across all moderate-and vigorous-intensity activities (10). To mitigate over-reporting, total PA was capped at 1,680 min per week per participant (equivalent to 4 hours of PA per day), consistent with ATLS computation guidelines (24). Within the ATLS analysis framework, participants were further classified by PA level as inactive (< 1,680 METs-min/week), minimally active (1,680–2,519 METs-min/week), or highly active (≥ 2,520 METs-min/week), consistent with ATLS guidelines for adolescents aged 14–19 years (24). This PA level classification serves to characterize the distribution of activity intensities within the full sample and is distinct from the binary grouping criterion (≥3 days/week VPA) used for group allocation, as described in the Participants and Recruitment section above.

### 2.6 Screen time and sleep duration

Sedentary behavior was defined as total daily recreational screen time, encompassing watching TV, playing video games, using a computer, and browsing the internet, assessed separately for weekdays and weekends. Total daily screen time was capped at 16 h per day, consistent with ATLS computation guidelines (24). Mean daily screen time was calculated, and participants were categorized as exceeding recommendations if their mean daily screen time exceeded 2 h per day, in accordance with widely accepted pediatric guidelines. Sleep duration was measured separately for weekdays and weekends. Mean daily sleep duration was calculated as a weighted average across both periods. Participants were classified as obtaining sufficient sleep if their mean daily sleep duration was ≥ 8 h per night (26).

### 2.7 Dietary habits

Dietary behavior was assessed using the ATLS questionnaire. This captured the frequency of consumption of 11 specific food and beverage items during a typical week (breakfast, vegetables, fresh fruit, dairy products, sugar-sweetened beverages, fast food, fried potatoes, bakery products, sweets and chocolate, and energy drinks). Response options ranged from 0 (never) to 7 days per week (daily). Dietary frequency was categorized into three groups: 1–2 days per week, 3–4 days per week, and ≥ 5 days per week.

### 2.8 Statistical analysis

All statistical analyses were performed using IBM SPSS Statistics (version 29; IBM Corp., Armonk, NY, United States). Continuous variables were presented as means and standard deviations (SD), and categorical variables as frequencies and percentages (%). Prior to inferential testing, the normality of all continuous variables was assessed using the Shapiro–Wilk test. For normally distributed continuous variables, between-group differences (physically active vs. physically inactive) were examined using an independent samples t-test; the Mann–Whitney U test was used as a non-parametric alternative for variables that violated the normality assumption. Categorical variables were compared between groups using the Chi-square (*χ*^2^) test, with Fisher’s exact test applied where expected cell frequencies were < 5. Effect sizes were calculated for all comparisons: Cohen’s d was used for continuous variables: values of 0.20, 0.50, and 0.80 interpreted as small, medium, and large effects, respectively (27); Cramér’s V was used for categorical variables: values of 0.10, 0.30, and 0.50 interpreted as small, medium, and large effects, respectively. The threshold for statistical significance was set at *p* < 0.05 for all analyses.

For requirements for a specific article type please refer to the Article Types on any Frontiers journal page. Please also refer to Author Guidelines for further information on how to organize your manuscript in the required sections or their equivalents for your field^1^.

## 3 Result

Table 1 (below) presents the anthropometric characteristics of both groups. The groups did not differ significantly in age (16.61 ± 0.63 vs. 16.66 ± 0.63 years; *p* = 0.835) or height (1.73 ± 0.07 vs. 1.75 ± 0.05 m; *p* = 0.155). Physically inactive participants had a significantly higher body weight (91.04 ± 22.71 vs. 72.56 ± 17.35 kg; *p* < 0.001; *d* = 0.931), BMI (30.28 ± 7.25 vs. 23.64 ± 5.39 kg/m^2^; *p* < 0.001; *d* = 1.060), waist circumference (97.35 ± 15.09 vs. 85.46 ± 12.61 cm; *p* < 0.001; *d* = 0.866), and WHtR (0.562 ± 0.090 vs. 0.488 ± 0.071; *p* < 0.001; *d* = 0.927) compared with physically active participants. These differences were both statistically significant and clinically meaningful, as indicated by larger effect sizes (*d* > 0.80) across all anthropometric indicators. According to IOTF criteria, 51.9% of physically inactive participants were classified as obese compared to 17.1% of physically active participants, and combined overweight and obesity was substantially more prevalent among physically inactive participants (77.8% vs. 38.6%; *χ*^2^ *p* < 0.001; Cramér’s V = 0.425). Abdominal obesity (WHtR ≥ 0.50) was evident in 72.2% of physically inactive participants vs. 31.4% of physically active participants (*p* < 0.001; Cramér’s V = 0.388).

**Table 1:** Anthropometric characteristics and obesity indicators by group.

Variable	Physically inactive (*n* = 54)	Physically active (*n* = 70)	*p*	*d*
Age (years)	16.61 ± 0.63	16.66 ± 0.63	0.835	0.073
Weight (kg)	91.04 ± 22.71	72.56 ± 17.35	<0.001*	0.931
Height (m)	1.73 ± 0.07	1.75 ± 0.05	0.155	0.259
BMI (kg/m^2^)	30.28 ± 7.25	23.64 ± 5.39	<0.001*	1.060
Waist circumference (cm)	97.35 ± 15.09	85.46 ± 12.61	<0.001*	0.866
WHtR	0.562 ± 0.090	0.488 ± 0.071	<0.001*	0.927
IOTF BMI category, *n* (%)			<0.001*	V = 0.425
Underweight	2 (3.7%)	5 (7.1%)		
Normal weight	10 (18.5%)	38 (54.3%)		
Overweight	14 (25.9%)	15 (21.4%)		
Obese	28 (51.9%)	12 (17.1%)		
Overweight + Obese	42 (77.8%)	27 (38.6%)		
WHtR category, *n* (%)			<0.001*	V = 0.388
Abdominal obesity (≥0.50)	39 (72.2%)	22 (31.4%)		
Normal (<0.50)	15 (27.8%)	48 (68.6%)		

Values are mean ± SD or *n* (%). * *p* < 0.05. d, Cohen’s d; V, Cramér’s V; IOTF, International obesity task force; WHtR = waist-to-height ratio.

Table 2 (below) presents PA outcomes. Physically active participants accumulated significantly greater total METs-min/week (4,084 ± 2,629 vs. 2,225 ± 2,980; *p* < 0.001; *d* = 0.667), moderate-intensity METs-min/week (835 ± 830 vs. 530 ± 897; *p* = 0.006; *d* = 0.355), vigorous-intensity METs-min/week (2,301 ± 2,174 vs. 1,295 ± 2,143; *p* < 0.001; *d* = 0.466), and total PA minutes per week (796 ± 479 vs. 422 ± 510; *p* < 0.001; *d* = 0.758) compared to physically inactive participants, with medium-to-large effect sizes across all variables. It should be noted that between-group differences in PA energy expenditure and PA level categories are partly expected given that the grouping variable itself was defined based on PA participation status; the primary analytical value of these comparisons lies in quantifying the magnitude of energy expenditure differential between groups rather than in the PA level categorization per se. PA level categorization indicated that 63.0% of physically inactive participants were inactive (< 1,680 METs-min/week) compared with 15.7% of physically active participants. Meanwhile, 68.6% of physically active participants were classified as *highly active* (≥ 2,520 METs-min/week) compared to only 24.1% of physically inactive participants (*χ*^2^ *p* < 0.001; Cramér’s V = 0.501).

**Table 2:** Physical activity outcomes by group.

Variable	Physically inactive (*n* = 54)	Physically active (*n* = 70)	*p*	*d*
Total METs-min/week	2,225 ± 2,980	4,084 ± 2,629	<0.001*	0.667
Moderate METs-min/week	530 ± 897	835 ± 830	0.006*	0.355
Vigorous METs-min/week	1,295 ± 2,143	2,301 ± 2,174	<0.001*	0.466
Total PA min/week (capped)	422 ± 510	796 ± 479	<0.001*	0.758
PA level category, *n* (%)			<0.001*	V = 0.501
Inactive (<1,680 METs-min/week)	34 (63.0%)	11 (15.7%)		
Minimally active (1,680–2,519 METs min/week)	7 (13.0%)	11 (15.7%)		
Highly active (≥2,520 METs-min/week)	13 (24.1%)	48 (68.6%)		

Values are mean ± SD or *n* (%). Total PA capped at 1,680 min/week per ATLS guidelines. * *p* < 0.05. d = Cohen’s d; V = Cramér’s V.

Table 3 (below) presents screen time and sleep outcomes. No statistically significant between-group differences were observed for any screen time or sleep variable. Mean daily screen time was comparable between physically inactive participants and physically active participants (2.18 ± 1.32 vs. 2.06 ± 1.38 h/day; *p* = 0.351; d = 0.091), and the proportion classified as having high screen time (> 2 h/day) was similar across groups (37.0% vs. 32.9%; *p* = 0.768; Cramér’s V = 0.026). Mean sleep duration also did not differ significantly (7.53 ± 1.20 vs. 7.09 ± 1.04 h/night; *p* = 0.191; *d* = 0.406), and the prevalence of insufficient sleep (< 8 h/night) was nearly identical between physically active and physically inactive participants (83.3% vs. 84.3%; *p* = 1.000; Cramér’s V = 0.000). Although the effect size for sleep duration approached a small-to-medium magnitude (*d* = 0.406), this comparison did not reach statistically significant and should be considered an exploratory observation only. Collectively, these findings suggest that screen time and sleep profile do not differ meaningfully between groups and were presented descriptively rather than as inferential conclusions.

**Table 3:** Screen time and sleep duration by group.

Variable	Physically inactive (*n* = 54)	Physically active (*n* = 70)	*p*	*d*
Screen time—weekdays (h/day)	2.26 ± 1.48	2.10 ± 1.67	0.149	0.103
Screen time—weekends (h/day)	1.96 ± 1.72	1.94 ± 1.74	0.874	0.011
Screen time—average (h/day)	2.18 ± 1.32	2.06 ± 1.38	0.351	0.091
Sleep—weekdays (h/night)	7.72 ± 1.28	7.20 ± 1.15	0.052	0.430
Sleep—weekends (h/night)	7.60 ± 1.31	7.38 ± 1.10	0.355	0.193
Sleep—average (h/night)	7.53 ± 1.20	7.09 ± 1.04	0.191	0.406
Screen time category, *n* (%)			0.768	V = 0.026
High (>2 h/day)	20 (37.0%)	23 (32.9%)		
Low (≤2 h/day)	34 (63.0%)	47 (67.1%)		
Sleep category, *n* (%)			1.000	V = 0.000
Sufficient (≥8 h/night)	9 (16.7%)	11 (15.7%)		
Insufficient (<8 h/night)	45 (83.3%)	59 (84.3%)		

Values are mean ± SD or *n* (%). Screen time capped at 16 h/day per ATLS guidelines. * *p* < 0.05. *d* = Cohen’s d; V = Cramér’s V.

Table 4 (below) presents dietary habit frequencies by group. Of the ten dietary behaviors assessed, only fresh fruit consumption differed significantly between groups. Physically active participants reported significantly higher weekly fresh fruit intake than participants inactive (5.44 ± 2.22 vs. 4.24 ± 2.43 days/week; *p* = 0.006; *d* = 0.520, medium effect). Physically active participants were more likely to consume fresh fruit five or more days per week (62.9% vs. 40.7%), while physically inactive participants were more likely to do so only 1–2 days per week (29.6% vs. 10.0%; *χ*^2^ *p* = 0.010; Cramér’s V = 0.272). Given number of dietary comparisons conducted, these isolated significant findings should be interpreted with caution, as it may reflect an inflated Type I error in the absence of multiple testing correction; it is presented as an exploratory finding rather than a confirmatory result. No significant differences between-group differences were evident for breakfast consumption, sugary beverages, vegetables, dairy products, fast food, fried potatoes, cakes and pastries, sweets and chocolate, or energy drinks (all *p* > 0.05; all Cramér’s V < 0.10).

**Table 4:** Dietary habit frequencies by group.

Dietary item	Category	Physically inactive (*n* = 54)	Physically active (*n* = 70)	Mean ± SD physically inactive	Mean ± SD physically active	*p*
Breakfast	1–2 days	9 (16.7%)	12 (17.1%)	5.63 ± 2.56	5.20 ± 2.64	0.434
	3–4 days	9 (16.7%)	19 (27.1%)			
	5 + days	36 (66.7%)	39 (55.7%)			
Sugary beverages	1–2 days	13 (24.1%)	20 (28.6%)	3.85 ± 2.08	3.94 ± 2.28	0.945
	3–4 days	28 (51.9%)	27 (38.6%)			
	5 + days	13 (24.1%)	23 (32.9%)			
Vegetables	1–2 days	10 (18.5%)	6 (8.6%)	5.15 ± 2.36	5.86 ± 2.17	0.096
	3–4 days	12 (22.2%)	14 (20.0%)			
	5 + days	32 (59.3%)	50 (71.4%)			
Fresh fruits*	1–2 days	16 (29.6%)	7 (10.0%)	4.24 ± 2.43	5.44 ± 2.22	0.006*
	3–4 days	16 (29.6%)	19 (27.1%)			
	5 + days	22 (40.7%)	44 (62.9%)			
Milk/dairy	1–2 days	7 (13.0%)	6 (8.6%)	5.39 ± 2.35	5.60 ± 2.26	0.770
	3–4 days	17 (31.5%)	20 (28.6%)			
	5 + days	30 (55.6%)	44 (62.9%)			
Fast food	1–2 days	20 (37.0%)	18 (25.7%)	3.54 ± 1.87	3.74 ± 1.83	0.407
	3–4 days	19 (35.2%)	32 (45.7%)			
	5 + days	15 (27.8%)	20 (28.6%)			
Fried potatoes	1–2 days	25 (46.3%)	22 (31.4%)	3.15 ± 1.73	3.43 ± 1.67	0.247
	3–4 days	18 (33.3%)	33 (47.1%)			
	5 + days	11 (20.4%)	15 (21.4%)			
Cakes/pastries	1–2 days	26 (48.1%)	24 (34.3%)	3.07 ± 2.14	3.13 ± 1.52	0.311
	3–4 days	15 (27.8%)	34 (48.6%)			
	5 + days	13 (24.1%)	12 (17.1%)			
Sweets/chocolate	1–2 days	24 (44.4%)	26 (37.1%)	3.26 ± 2.05	3.27 ± 1.86	0.832
	3–4 days	13 (24.1%)	28 (40.0%)			
	5 + days	17 (31.5%)	16 (22.9%)			
Energy drinks	1–2 days	46 (85.2%)	57 (81.4%)	1.52 ± 1.14	1.61 ± 1.18	0.551
	3–4 days	6 (11.1%)	10 (14.3%)			
	5 + days	2 (3.7%)	3 (4.3%)			

Values are *n* (%) for categories and mean ± SD for weekly frequency (0–7 days). * Significant difference between groups (*p* < 0.05). *p*-values based on Mann–Whitney *U* test for continuous comparisons. The fresh fruit finding should be considered exploratory given the number of comparisons conducted.

## 4 Discussion

This study examined obesity-related risk behaviors between physically active and physically inactive male Saudi adolescents in Riyadh using the validated ATLS questionnaire, drawing on SCT as a conceptual lens. Three principal findings emerged: (i) physically inactive participants exhibited significantly higher BMI, waist circumference, and WHtR values than physically active participants, with large effect sizes across all anthropometric indicators; (ii) physically active participants accumulated substantially greater PA energy expenditure (METs-min/week); (iii) among the ten dietary behaviors assessed, only fresh fruit consumption differed significantly between groups. Screen time and sleep duration did not differ meaningfully between groups.

The most clinically significant finding was the between-group anthropometric divergence. Physically inactive participants had a mean BMI of 30.28 kg/m^2^, placing the group in the obese range, versus 23.64 kg/m^2^ among physically active participants, with 51.9% of physically inactive participants classified as obese by IOTF criteria and 77.8% as overweight or obese combined, compared to 17.1 and 38.6% of physically active participants, respectively. These findings were consistent with research findings from Saudi Arabia and GCC countries that document an elevated prevalence of obesity among physically inactive adolescents (7, 8, 25, 28), and with global evidence confirming that higher BMI was inversely associated with MVPA levels across adolescent populations (29–31). Abdominal obesity (WHtR ≥ 0.50) was present in 72.2% of physically inactive participants compared with 31.4% of physically active participants (*d* = 0.927). The WHtR is a population-independent indicator increasingly used to detect abdominal adiposity irrespective of age or sex-specific norms (32, 33); the large between-group difference in the current results underscores the elevated cardiometabolic risk profile of physically inactive participants. Conceptually, within the SCT framework, these disparities may reflect differences in exercise-related self-efficacy, outcome expectations, and behavioral capability between groups (12, 34). In short, physically active participants may possess higher exercise-related self-efficacy and greater confidence in their capacity to perform and sustain regular PA, which physically inactive participant may hold lower outcome expectations and perceive fewer benefits from regular PA engagement. However, it should be noted that these constructs were not directly measured in this study, and these interpretations therefore represent theoretically informed speculation rather than empirically derived conclusions. Importantly, given the cross-sectional design, reverse causation cannot be excluded, adolescents with obesity may engage less in PA due to pre-existing obesity-related physical limitations or psychosocial barriers rather than inactivity necessarily preceding or causing obesity (15, 35).

Physically active participants accumulated nearly twice the total METs-min/week of physically inactive participants (4,084 vs. 2,225; *d* = 0.667), and nearly double the total PA minutes per week (796 vs. 422; *d* = 0.758), with 68.6% of physically active participants classified as highly active compared to 24.1% of physically inactive participants. Conversely, 63.0% of physically inactive participants were inactive compared with 15.7% of physically active participants. These inactivity rates align with global evidence that approximately 80% of adolescents fail to meet the WHO’s recommendation of 60 min of MVPA per day (36) and with regional data documenting widespread inactivity among Saudi adolescents (9, 37). This result also concurs with a school-based study confirming that physically inactive adolescents were 1.7–1.8 times more likely to be overweight or obese than physically active peers (38). Conceptually, within the SCT framework, structural features of Riyadh environment, including car dependency, extreme heat, and limited pedestrian infrastructure, may represent environmental determinants that through reciprocal determinism, suppress behavioral capability and self-efficacy for PA engagement among physical inactive adolescents (10, 21, 34). These were plausible explanations informed by the SCT framework; however, these environmental factors were not directly measured in this study and should therefore be regarded as possible rather than established mechanisms. The SCT construct of observational learning may also be relevant: adolescents who regularly observe peers and family members engaging in structured PA may be more likely to develop positive exercise-related outcome expectations and higher exercise-related self-efficacy (13, 16). Peer-based programs leveraging observational learning and social reinforcement may therefore represent a theoretically grounded approach to increasing PA participation among physically inactive Saudi adolescents, though the current study design does not permit conclusions regarding the effectiveness of such interventions.

No significant between-group differences were evident for screen time (2.18 ± 1.32 vs. 2.06 ± 1.38 h/day; *p* = 0.351) or sleep duration (7.53 ± 1.20 vs. 7.09 ± 1.04 h/night; *p* = 0.191). Screen-based sedentary behavior appears to be a near-universal norm among Saudi adolescents regardless of PA status (25), consistent with evidence that PA and screen time operate as partially independent behavioral dimensions rather than mere opposites (39, 40). One possible conceptual explanation, drawn on the SCT framework, is that social support from family and peer networks may exert a stronger normalizing influence on screen-based behaviors than PA participation status; however, social support was not measured in this study and this interpretation is speculative. Insufficient sleep (< 8 h/night) was nearly universal in both groups (83.3 and 84.3%), substantially exceeding Western estimates and consistent with prior Saudi data (11). Evidence suggested that concurrent high PA and low screen time may be simultaneously required to meaningfully improve sleep adequacy among adolescents (41). The uniform sleep insufficiency across-group implicates structural and sociocultural drivers, such as late-night screen use and academic pressures that may operate independently of PA status and require population-wide rather than PA-targeted intervention, though these drivers were not directly assessed in current study.

Of the ten dietary items, only fresh fruit consumption differed significantly between groups (5.44 ± 2.22 vs. 4.24 ± 2.43 days/week; *p* = 0.006; *d* = 0.520). Given the number of dietary comparisons conducted, this isolated finding should be interpreted with caution as it may reflect an inflated Type I error in the absence of multiple testing correction; it is therefore presented as an exploratory observation. Notwithstanding this caveat, the finding was consistent with evidence that PA participation and healthier dietary behaviors tend to cluster through shared self-regulatory capacity (42). Conceptually, this may be underscore through the SCT construct of reciprocal determinism, whereby PA engagement may reinforces health-oriented behavioral dispositions more broadly, including dietary vigilance; however, self-regulation capacity and dietary-related SCT constructs were not measured in current study (12, 34). Both groups reported equally high consumption of sugary beverages (3.94 ± 2.28 vs. 3.85 ± 2.08 days/week) and fast food (3.74 ± 1.83 vs. 3.54 ± 1.87 days/week), suggesting that PA participation alone may not be sufficient to protect against obesogenic dietary patterns in environments where energy-dense food are normalized and easily accessible (35, 43). These findings were consistent with evidence that personal cognitive SCT constructs have limited predictive utility for dietary behavior in collectivist, family-oriented food environments (36), though this explanation remains theoretical given that dietary-related SCT constructs were not assessed. The observed patterns reflects the well-documented nutrition transition in Saudi Arabia and underscores the need for multi-level interventions that address the broader food environment alongside individual level strategies.

The findings of this study have several public health implications for Saudi Arabia and similar GCC contexts, though it is important to acknowledge that the cross-sectional design does not permit causal inference and the convenience sample from one city limits generalizability; findings should therefore be interpreted cautiously in context of policy development. First, the markedly higher prevalence of obesity and abdominal adiposity among physically inactive participants, structured school-based PA programs initiatives may be represent a promising avenue for intervention, with program design potentially informed by SCT contracts such as exercise self-efficacy and social reinforcement (34, 35). Second, the similarly poor screen time and sleep profiles across both groups suggest that PA-targeted interventions alone may be insufficient, and that comprehensive school health programs simultaneously addressing screen time management and sleep hygiene may also be warranted. Third, the near-identical across-group obesogenic dietary patterns highlight the potential value of food environment interventions, such as healthier school canteen policies, restricting the availability of sugar-sweetened beverages on school premises, and nutrition literacy programs, to complement individually focused behavioral dietary strategies. Finally, Vision 2030, which explicitly prioritizes sport participation, physical fitness, and healthy living as national development goals, provides a favorable policy environment for scaling evidence-based PA promotion and obesity prevention programs targeting adolescents (44), though the effectiveness of specific interventions cannot be inferred from current study design.

This study has several strengths: it employs the validated, culturally appropriate ATLS questionnaire with a comparative design informed by SCT, uses multiple obesity indicators (BMI via IOTF and WHtR), rigorously applies ATLS computation caps, and reports effect sizes throughout. Despite this, the current study is subject to several limitations. First, cross-sectional design precludes the assumption of causal inference and reverse causation cannot be excluded adolescents with obesity may participate less in PA due to pre-existing physical limitations rather than inactivity preceding obesity. Second, recruiting a male-only convenience sample from public high schools in Riyadh limits generalizability to female adolescents, private school students, and those from other regions. Third, its reliance on self-reported data introduced potential measurement bias. Fourth, important sociodemographic variables, including socioeconomic status, parental education, and household income, were not collected, precluding multivariable adjustment for potential cofounders. Fifth, and critically, although the current study is theoretically framed around SCT, no SCT constructs, including self-efficacy, outcome expectations, observational learning, or social support, were directly measured; this represents a fundamental disconnect between the theoretical framing and empirical methodology, meaning all SCT-based interpretations in current study were conceptual rather than empirically derived, and formal mediation or pathway testing of the SCT framework was not possible. Given the above, future research should incorporate device-based PA measurement via accelerometry, include female adolescents to address the documented gender disparities in PA participation (45), include female adolescents, directly assess SCT constructs, collect sociodemographic covariate to enable multivariable adjustment, and adopt longitudinal designs to establish causal pathways between PA participation and obesity risk.

## 5 Conclusion

Physical activity participation was associated with lower obesity risk among male Saudi adolescents, as evidenced by the large between-group differences in BMI, waist circumference, WHtR, and IOTF-classified obesity prevalence; however, given the cross-sectional design, these associations should be interpreted cautiously as they cannot establish temporal directionality or causal inference. Physically active participants also accumulated significantly greater physical activity energy expenditure and reported higher fresh fruit consumption. Screen time and sleep values were similarly unfavorable across both groups, suggesting the pervasive influence of an obesogenic environment irrespective of PA status. These findings highlight the potential value of PA promotion, dietary reform, and screen time and sleep initiatives for this population. Longitudinal research directly measuring SCT constructs, incorporating device-based PA assessment, including female adolescents, and accounting for sociodemographic confounders is needed to establish causal pathways and inform evidence-based public health policy for adolescent obesity in Saudi Arabia.

## Data Availability

The raw data supporting the conclusions of this article will be made available by the authors, without undue reservation.

## Footnotes

1 For Original Research articles, please note that the Material and Methods section can be placed in any of the following ways: before Results, before Discussion or after Discussion.
